# Supplementary material for: Regulation of chromatin modifications through coordination of nucleus size and epithelial cell morphology heterogeneity
Source: Commun Biol. 2025 Feb 20;8:269. doi: 10.1038/s42003-025-07677-w (PMC11842846; doi:10.1038/s42003-025-07677-w)
Supplement: Supplementary file 3 — Description of Additional Supplementary File [file 42003_2025_7677_MOESM3_ESM.pdf]

Description of Additional Supplementary File

File name: Supplemental data 1.zip
Description: Contains the source data necessary for reproducing all plots presented in the manuscript.

File name: Supplemental data 2.zip
Description: Includes the raw data used for the multivariable analysis.
