# Supplementary material for: Regulation of chromatin modifications through coordination of nucleus size and epithelial cell morphology heterogeneity
Source: Commun Biol. 2025 Feb 20;8:269. doi: 10.1038/s42003-025-07677-w (PMC11842846; doi:10.1038/s42003-025-07677-w)
Supplement: Supplementary file 6 — reporting-summary [file 42003_2025_7677_MOESM6_ESM.pdf]

Corresponding author(s): Neil Lin

Last updated by author(s): Dec 11, 2024

Reporting Summary

Nature Portfolio wishes to improve the reproducibility of the work that we publish. This form provides structure for consistency and transparency in reporting. For further information on Nature Portfolio policies, see our Editorial Policies and the Editorial Policy Checklist.

Statistics

For all statistical analyses, confirm that the following items are present in the figure legend, table legend, main text, or Methods section.

n/a	Confirmed	
☐	☒	The exact sample size (*n*) for each experimental group/condition, given as a discrete number and unit of measurement
☐	☒	A statement on whether measurements were taken from distinct samples or whether the same sample was measured repeatedly
☐	☒	The statistical test(s) used AND whether they are one- or two-sided<br>*Only common tests should be described solely by name; describe more complex techniques in the Methods section.*
☒	☐	A description of all covariates tested
☒	☐	A description of any assumptions or corrections, such as tests of normality and adjustment for multiple comparisons
☐	☒	A full description of the statistical parameters including central tendency (e.g. means) or other basic estimates (e.g. regression coefficient) AND variation (e.g. standard deviation) or associated estimates of uncertainty (e.g. confidence intervals)
☐	☒	For null hypothesis testing, the test statistic (e.g. *F*, *t*, *r*) with confidence intervals, effect sizes, degrees of freedom and *P* value noted<br>*Give P values as exact values whenever suitable.*
☒	☐	For Bayesian analysis, information on the choice of priors and Markov chain Monte Carlo settings
☒	☐	For hierarchical and complex designs, identification of the appropriate level for tests and full reporting of outcomes
☐	☒	Estimates of effect sizes (e.g. Cohen's *d*, Pearson's *r*), indicating how they were calculated

Our web collection on statistics for biologists contains articles on many of the points above.

Software and code

Policy information about availability of computer code

Data collection	No software was used.
Data analysis	Cellpose, Matlab (R2022a), ImageJ (1.54i), and Prism Graphpad (10) were utilized for analysis.

For manuscripts utilizing custom algorithms or software that are central to the research but not yet described in published literature, software must be made available to editors and reviewers. We strongly encourage code deposition in a community repository (e.g. GitHub). See the Nature Portfolio guidelines for submitting code & software for further information.

Data

Policy information about availability of data

All manuscripts must include a data availability statement. This statement should provide the following information, where applicable:

- Accession codes, unique identifiers, or web links for publicly available datasets
- A description of any restrictions on data availability
- For clinical datasets or third party data, please ensure that the statement adheres to our policy

All data and codes are available from the corresponding author upon request.

Research involving human participants, their data, or biological material

Policy information about studies with human participants or human data. See also policy information about sex, gender (identity/presentation), and sexual orientation and race, ethnicity and racism.

Reporting on sex and gender	N/A
Reporting on race, ethnicity, or other socially relevant groupings	N/A
Population characteristics	N/A
Recruitment	N/A
Ethics oversight	N/A

Note that full information on the approval of the study protocol must also be provided in the manuscript.

Field-specific reporting

Please select the one below that is the best fit for your research. If you are not sure, read the appropriate sections before making your selection.

☒ Life sciences ☐ Behavioural & social sciences ☐ Ecological, evolutionary & environmental sciences

For a reference copy of the document with all sections, see nature.com/documents/nr-reporting-summary-flat.pdf

Life sciences study design

All studies must disclose on these points even when the disclosure is negative.

Sample size	Sample size was not predetermined due to resource constraints to perform higher number of experiments and our past experiences that sample numbers in this range can reliably detect differences. We have replicated experiments at least 3-5 times.
Data exclusions	Cells that form doming structures were excluded from analysis, as these cells do not represent jammed epithelial cells.
Replication	All results were reproducible.
Randomization	Cells utilized for control and treated cells were chosen at random.
Blinding	Blind analysis was performed.

Reporting for specific materials, systems and methods

We require information from authors about some types of materials, experimental systems and methods used in many studies. Here, indicate whether each material, system or method listed is relevant to your study. If you are not sure if a list item applies to your research, read the appropriate section before selecting a response.

Materials & experimental systems

n/a	Involved in the study	
☐	☒	Antibodies
☐	☒	Eukaryotic cell lines
☒	☐	Palaeontology and archaeology
☐	☒	Animals and other organisms
☒	☐	Clinical data
☒	☐	Dual use research of concern
☒	☐	Plants

Methods

n/a	Involved in the study	
☒	☐	ChIP-seq
☒	☐	Flow cytometry
☒	☐	MRI-based neuroimaging

Antibodies

Antibodies used	The primary antibodies utilized were H3K27me3 (9733S, lot 19, Cell Signaling Technologies, mAb) , H3K9ac (61663, lot 09919001, Active Motif, mAb), Ezh2 (3147S, lot 5, Cell Signaling Technologies, mAb), and UTX (33510S, lot 4, Cell Signaling Technologies, mAb).

Validation	All antibodies have been validated by the manufacturer, as noted on the product website. In addition, several citations of published works are available for each antibody.

Eukaryotic cell lines

Policy information about cell lines and Sex and Gender in Research

Cell line source(s)	The MDCK II cell line was a gift from Jeffrey Fredberg at Harvard University. The HaCat cells were obtained from AddexBio, catalog number T0020001.
Authentication	Cell lines were not authenticated.
Mycoplasma contamination	Cell line tested negative for Mycoplasma.
Commonly misidentified lines (See ICLAC register)	No commonly misidentified cell line was used in this study.

Animals and other research organisms

Policy information about studies involving animals; ARRIVE guidelines recommended for reporting animal research, and Sex and Gender in Research

Laboratory animals	Mus musculus embryos with the genotype K14Cre; R26mTmG at embryonic day 11.5 and 12.5 were used.
Wild animals	No wild type animals were used
Reporting on sex	Embryos were not sexed and were selected at random in all experiments.
Field-collected samples	N/A
Ethics oversight	All experiments involving mice were approved by the UCLA Institutional Animal Care and Use Committee (Protocol Number ARC-2019-013)

Note that full information on the approval of the study protocol must also be provided in the manuscript.

Plants

Seed stocks	N/A
Novel plant genotypes	N/A
Authentication	N/A
